# The factors involved in surgical decision-making in younger women diagnosed with breast cancer in Aotearoa New Zealand: A qualitative analysis

**DOI:** 10.1177/13591053241237075

**Published:** 2024-03-08

**Authors:** Tania Blackmore, Kimberley Norman, Vanessa Burrett, Jenni Scarlet, Ian Campbell, Ross Lawrenson

**Affiliations:** 1 University of Waikato, New Zealand; 2 Te Whatu Ora – Health New Zealand Waikato, New Zealand

**Keywords:** breast cancer, qualitative research, shared decision-making, surgery, younger women

## Abstract

Women diagnosed with breast cancer must make important surgical decisions. The decision-making process for younger women is complex, with this group more likely to have an advanced diagnosis and life-stage considerations that can impact on treatment. This study investigated the decision-making process of women aged <50 years who had undergone breast cancer surgery within the preceding 12 months in Aotearoa New Zealand. Twelve women participated in semi-structured qualitative interviews to explore the factors that influenced treatment decisions. Thematic analysis resulted in three themes. Fear was the main concept identified as the primary influence on initial decision-making. Good quality shared decision-making between patient and clinician was found to provide essential support during the diagnosis to treatment period. In addition, women expressed a need for multi-modal presentation of medical information and more material reflecting younger women. These findings inform provision for younger women making surgical decisions when diagnosed with breast cancer.

## Introduction

Breast cancer (BC) affects millions of women worldwide and is the most common cancer to impact women in Aotearoa New Zealand (NZ), with over 3000 women and around 25 men diagnosed in 2019 (Ministry of Health, 2021). The median age of diagnosis of invasive BC is 58 years (Breast Cancer Foundation NZ, 2022). While many women are diagnosed following breast screening, a proportion of younger women who are under NZs screening age range of 45–69 are diagnosed each year. For example, for the period 2003–2019, 13.4% of NZ women diagnosed with invasive BC were aged 19–44 (Breast Cancer Foundation NZ, 2022). BC in younger women is more likely to be advanced, with aggressive tumour presentation (La et al., 2019). Māori are the Indigenous peoples of NZ, and wāhine Māori (Māori women) are more likely to be diagnosed with high-risk BC than non-Māori and tend to be diagnosed at a younger age, with 17% of wāhine Māori aged <45 at diagnosis, compared to 11% of NZ women of European descent (NZE) across the same time period (Breast Cancer Foundation NZ, 2022). Māori are also more likely to undergo mastectomy than NZE women (Campbell et al., 2018; Breast Cancer Foundation NZ, 2022).

Surgical treatment for early-stage BC involves either breast conserving surgery (BCS) (also called wide local excision or lumpectomy) which removes the cancer with a margin while sparing most of the breast tissue (Sun et al., 2021), or mastectomy (removal of the breast). Mastectomy may be performed with, or without reconstructive surgery (Chang et al., 2016). Breast reconstruction aims to restore breast form and improve post-surgery body image and improve quality of life (Clarke et al., 2021). However, breast reconstruction comes with further surgical decisions, including the type of reconstruction (implants or autologous tissue) (Holland et al., 2016) and whether to undergo major surgery, with attendant greater risk of complication and need for more surgery in future. In defiance of social and cultural norms, some women are increasingly considering the option to ‘go flat’, that is, to not reconstruct the breast following mastectomy (Morrison and Karp, 2022; Webb et al., 2019). However, there is some evidence from the United States that this option is not commonly discussed with women during the decision-making period (Wakeley et al., 2020).

The surgical decision-making process for younger women diagnosed with BC is particularly complex (Rosenberg et al., 2018) and varies from that of older women (Recio-Saucedo et al., 2018). Younger women must balance the distress of a cancer diagnosis with life-stage, age-related issues, including fertility (Rosenberg et al., 2018), sexuality, relationships and body image (Paterson et al., 2016). An increasing trend in the United States for younger women to choose bilateral prophylactic mastectomy (Rosenberg et al., 2019) has been linked to fear-driven decision-making in this group (Gu et al., 2018). Women diagnosed with BC at a younger age are also more likely to carry a hereditary BC gene mutation and are more likely to opt for reconstruction of the breast following mastectomy, a trend that has been reported both internationally (Soon et al., 2019) and within NZ (Breast Cancer Foundation NZ, 2022). Given the complexities younger women face, they have high information needs (Recio-Saucedo et al., 2018) and are more at risk of experiencing anxiety and depression following a BC diagnosis (Campbell-Enns and Woodgate, 2017). Important treatment decisions also typically need to be made within a short timeframe following diagnosis (Maes-Carballo et al., 2021). Therefore, it is important to provide younger women diagnosed with BC with appropriate levels of support as they approach treatment decisions.

Shared decision-making between patient and clinician aims for a collaborative discussion of treatment options that considers both patient preference and clinical indications (Grabinski et al., 2018). Subsequently, the shared decision-making process has been recommended in clinical practice for BC (Gennari et al., 2021; Mann et al., 2022). Shared decision-making can help to mitigate the fear and anxiety associated with surgical decisions (Blackmore et al., 2024) and can lead to positive patient outcomes (Clarke et al., 2021). It should be noted that, corresponding with the highly individual nature of how a BC diagnosis is received, not all patients are ready to engage in shared decision-making (Darmonkow et al., 2021). For example, some patients may continue to prefer physician-led decisions regarding certain aspects of their treatment, such as chemotherapy or radiation (Feiten et al., 2022). Younger BC patients, however, are more likely to be active in their health decisions (Recio-Saucedo et al., 2018; Sun et al., 2021) so shared decision-making may be an important aspect of their BC care.

While decision-making in BC is well-researched internationally, factors influencing decision-making has not been investigated in a cohort of NZ women diagnosed with BC. Therefore, this study explored the decision-making process of women aged <50 years who had undergone any form of BC surgery within the preceding 12 months. We aimed to discover the factors involved in women’s choice of the form of BC surgery, and how they had decided to undergo either BCS or mastectomy, with or without breast reconstruction.

## Methods

### Participant recruitment

This qualitative research is part of a larger explanatory sequential mixed method design (Curry and Nunez-Smith, 2015), which consisted of two parts. The quantitative section of this research (the explanatory component) was conducted first, followed by the qualitative section (the sequential component), which aimed to further elaborate on the quantitative study (Ivankova et al., 2006; Tariq and Woodman, 2013) to form a comprehensive view of the phenomenon studied. Therefore, participants in this study had been surveyed as part of a preceding quantitative study (unpublished data), which aimed to assess women’s satisfaction with their breast surgery using the BREAST-Q survey. This prior study involved 137 women with a confirmed stage I-II BC diagnosis who had received breast surgery within the previous 12 months. Participants were recruited through patient lists obtained from the former Waikato District Health Board (DHB), which had a patient population of 400,000+ but is now incorporated into a nationwide organisation called Te Whatu Ora. Women were invited to participate in the survey via anonymous text message providing the BREAST-Q survey link. The survey link provided access to a digital consent form, but patients could also ask for the study information and consent form to be mailed. Following the quantitative survey, women were asked to indicate further consent to be contacted for qualitative interview. Of those who consented to the qualitative stage, we purposively sampled women in the 30–50 age range. This range was chosen because the majority of BC occur in women aged over 50 in NZ (Breast Cancer Foundation NZ, 2024). Of those who had provided further verbal (via telephone) or written (via completed consent form or email) consent to interview, 12 women were eligible for inclusion in the current study. Ethical approval for the study was granted by the University of Waikato Human Research Ethics Committee (HREC (Health)2021#78). This research was supported by a Health Research Council of New Zealand Career Development Grant, HRC Ref: 21/961.

### Data collection

The 12 eligible women were contacted by phone or email, provided with further details of the interview stage and invited to participate. All participants were given a participant information sheet prior to interview and any questions or concerns were answered by the researcher. All 12 participants volunteered to take part and a suitable time and interview location were arranged. Interviews were initially carried out in the participant’s home (*n* = 4), but following COVID-19 restrictions, the remainder were carried out via Zoom (*n* = 2) or telephone (*n* = 6). Interviews were conducted from January to March 2022. While written consent had already been obtained from the quantitative survey, additional verbal consent was also obtained and recorded via audio recording device prior to the start of every interview.

Interviews were semi-structured and followed an interview guide, starting with women’s thoughts about their surgery, their shared decision-making experience, and whether they were provided with, and understood information about their diagnosis. The interview guide was developed by the research team, which ensured Māori cultural considerations were accommodated. The open-ended questions ensured that participants could direct the conversation to concepts that were significant to them, enabling nuanced experiences of BC surgery to be collected. However, this sample group was post-surgery, therefore decision-making had already occurred. Thus, the interview guide included questions focused specifically on the ‘decision-making process’ for each participant. The context in which BC is diagnosed and surgery is engaged with is considered positivist and operates with ‘shared decision-making’ as best practice in this environment. Therefore, the interview guide also included open-ended questions that related to the ‘shared decision-making’ concept. Open-ended questions included ‘please tell me about the factors that were important to you when making your decision to have surgery after you were diagnosed?’, ‘please tell me about anything that was important to you that you think may have influenced your decision to have surgery?’ and ‘please tell me about your experience with your surgeon when making your decision?’.

The aim of the study was restated prior to the start of each interview and participants were reminded of confidentiality and their right to withdraw at any time. Māori participants were given the option to begin the interview with a karakia (prayer), whakawhanaungatanga (the process of establishing relationships) and to include whānau (family) in interviews. All participants were thanked for their participation. Interviews were conducted by two NZ European female researchers and lasted between 30 and 120 minutes. Interviews were audio recorded and transcribed using Otter.ai (online transcription service). All transcripts were analysed collaboratively between the interviewers (TB and KN) and transcripts for Māori participants were reviewed by Māori researchers (VB and KT). The right to amend or review transcripts was offered to all participants; however, no participants requested a review or redaction of their interview.

### Analysis

All interview data was analysed using reflexive thematic analysis as outlined by Braun and Clarke (2006). This approach utilised a six-step process: (i) familiarisation of the data, (ii) coding, (iii) generating initial themes, (iv) developing and reviewing themes, (v) refining, defining and naming themes and (vi) write up (Clarke and Braun, 2013). Firstly, all transcripts were read, and re-read by two researchers (TB and KN) with a view to facilitate immersion in the data. Transcripts of participants identifying as Māori were reviewed by Māori researchers (VB and KT) The analysis took an inductive approach whereby the aim was to explore any concepts related to ‘shared decision-making’ or ‘decision-making’ for participants. In the left-hand margin of each transcript, sections of conversation that reflected these concepts were highlighted and coded. This study acknowledges that decision-making is a nuanced endeavour with no two women experiencing BC decision making the same. As such, in the right-hand margin of transcripts, any additional ideas and concepts that were significant to each participants’ discourse were highlighted and labelled, permitting new or unexpected concepts to be coded. Each transcript was analysed in turn, and then comparatively re-analysed for missing codes. All codes were listed, and redundant or repeated codes were removed. Code lists were discussed by all researchers and an initial six preliminary themes were generated. Resulting themes were discussed with the research team to ensure a reflexive, culturally inclusive and rigorous analysis process. This team included Māori and non-Māori, female and male, clinical and non-clinical academics and early to mid-career researchers. Both Māori and non-Māori researchers worked together to ensure the voices of Māori were not mis-interpreted or mis-represented from interview guide development to write up. Whilst Braun and Clarke (2021) highlight that the ability to achieve data saturation is situated and subjective, this analysis found no new themes were being interpreted when revisiting the transcripts and reflecting on codes already identified. The final three themes are detailed below.

## Results

Table 1 shows the characteristics of the 12 participants interviewed. Five (41.7%) women had received BCS and seven of the 12 (58.3%) had undergone mastectomy. Of those who underwent mastectomy, four (57.1%) had breast reconstruction and three (42.9%) did not reconstruct their breast following mastectomy.

**Table 1.** Participant characteristics (*N* = 12).

	*n*	%
Age group		
<40	2	16.7
40–45	5	41.7
46–50	5	41.7
Ethnicity		
New Zealand European	7	58.3
Māori	3	25.0
Other (European)	2	16.7
Surgery type		
BCS	5	41.7
Mastectomy alone	3	25.0
Breast reconstruction	4	33.3

The thematic analysis resulted in the following three themes: (1) influences on decision-making, (2) shared decision-making and (3) information and decision-making.

### Influences on decision-making

The concepts each woman associated with their breasts was notably a factor that influenced the decision to opt for surgery. Some women linked their BC to concepts including ‘disease’ that was recognised as a threat to survival. Fear was the main concept identified as a primary influence on initial decision-making. For some women, there was an urgency to remove the cancer from their body and for others, breasts were associated with functionality.

For many women, once their breast/s had been associated with cancer and the concept of ‘disease’, the decision to have a mastectomy and remove their breast became about survival. As two participants described:

For me, it was just a no brainer. I choose life over boobs. BC20, age 42, Māori, Mastectomy alone

As long as I was alive. That’s the main goal. BC23, age 44, Māori, Mastectomy and implant reconstruction

For some, being around for their family in the future was a significant influence over their surgical decision and surpassed the loss of their breast. Upholding personal responsibilities that are bigger than oneself, such as being a parent were important factors for survival, as one participant described:

I’ve got her [daughter] to worry about, so for me losing a breast and being alive to see her grow up is more important to me than having bits chopped out of my breast. BC22, age 45, non-Māori, Mastectomy alone

It was also interesting to hear that cosmetic appearance was not reported as a primary concern for this group. Although cosmesis was on their mind, ‘survival’ through taking surgical action against the cancer was positioned as more important:

Yes it [surgical outcome] was a worry. But again, it was just like, just get it out of me. And I’ll deal with that later’. BC12, age 42, non-Māori, BCS

The desire to have the ‘disease’ removed was significant to the decision-making process for both Māori and non-Māori women. The idea of ‘getting it gone’ was linked to concepts of survival, as described by two women:

I knew the most extreme measure of getting rid of that tissue would give me the best chance of surviving. So that was kind of like the big, the big tick, like yes! BC20, age 42, Māori, Mastectomy alone

Once you know that you have this thing growing in you, you just want it out. So, for me it was a no brainer. BC22, age 45, non-Māori, Mastectomy alone

The desire to get the cancer ‘gone’ was similarly expressed by those who had their breast conserved rather than removed, as one woman highlighted:

When you have that diagnosis, all you want to do is get it out of your body. Well, for me, it was just, just get it out. BC12, age 42, non-Māori, BCS

For some women, breasts were associated with a functional purpose, and the decision to have them removed was shaped by whether they were determined to have ‘served their purpose’. As one woman highlighted:

Well from my perspective, anyway, is that you know, my boobs have done their job. They’ve breastfed my two children. BC20, age 42, Māori, Mastectomy alone

Some women experienced the additional complexity of deciding whether to undergo breast reconstruction. Despite often being associated with a ‘functional’ purpose, some women expressed that the idea of living without their breast elicited feelings of fear. The concept of not having ‘something there’ in the space their removed breast once held was a driving factor in opting for immediate reconstructive surgery, as described by one woman:

I had tried to visualize what it would be like waking up after surgery without a breast, living without a breast before it got done. And like that pretty much scared me. So yeah, I was really pleased that I was offered it on the day. BC46, age 47, non-Māori, Mastectomy and DIEP reconstruction

Age was also a consideration for some women, who felt that they were too young to not undergo reconstruction. Some women discussed options with friends and family, indicating that influences and information on the decision-making process can come from sources outside an evidence-based positivist (medical) context. One woman detailed that after discussing it with friends and family she thought:

Well why not? You know, I’m not, I’m still relatively young, you know, why not just do it? BC43, age 48, non-Māori, Mastectomy and DIEP reconstruction

Others felt that they were too young to not have ‘something there’:

But I felt even at 44, I was still too young not to have something there. I knew that it may become a regret later on, if I didn’t do the reconstruction straightaway. BC23, age 44, Māori, Mastectomy and implant reconstruction

However, one of the youngest interviewees exemplified the additional struggle women face when contemplating reconstruction, and that some women have restricted decision-making options. She described her initial expectation of having reconstruction, contending with her surgeon not recommending the procedure and then finally trying to come to terms with the fact that she may not get reconstructive surgery:

But obviously it wasn’t really an option for me, at the end of the day. I mean, I could have had reconstruction, I could have had the reconstruction using my own tissue that would, it would have worked. It wouldn’t have been, it would have been quite a, a difficult path to go down. Yeah, so one of the things also was, at first, I didn’t really accept that I wasn’t going to have reconstruction. BC42, age 39, non-Māori, Mastectomy alone

Finally, for women who did have options for decision-making, influencing factors included not only the surgeon’s clinical advice, but also the advice from close friends and family who had lived experiences of BC. Some women valued the advice given by their surgeon, and also considered the clinical presentation of their disease when making a surgical decision. As one woman highlighted, her tumour was small and so felt it did not warrant a mastectomy:

I’ve got like, a tumour the size of that [indicates small size], like, why the hell would I? BC07, age 45, non-Māori, BCS

Another woman detailed how she checked her own research about options and went with her consultant’s advice:

Like mastectomy was just absolutely not needed. Because mine is pre or early breast cancer. BC32, age 49, non-Māori, BCS

For many women, the experience of witnessing close friends and family go through BC was a strong factor influencing decision-making. While cancer is a notably individualised experience, for some women the experiences they were exposed to through friends and family could impact decision-making. For one woman, watching her mother battle cancer recurrence following BCS, coupled with her mother’s advice on treatment was key to her treatment decision:

She [mother] always said, I wish I just had of had both my boobs off. So as part of my decision, it was no, I’ve just spent 30 years of living in the hospital with my mother. . ..I don’t want that for myself. So, I want, I want everything gone. BC10, age 50, non-Māori, Mastectomy and implant reconstruction

### Shared decision-making

Most women reported a sense of agency and participation in shared decision-making. Other women were happy to be primarily influenced by their surgeon’s recommendation. Shared decision-making was not perceived to be effectively achieved in only a minority of participant narratives.

Working with the surgeon as part of a shared decision-making process with a minimal power imbalance was positioned as positive to the overall BC experience, as highlighted by one woman:

They [surgical team] were doing it [decision making] with me, not to me. BC22, age 45, non-Māori, Mastectomy alone

One woman stressed the positivity of having a very patient-centred surgeon work with her, which was particularly important to her as a Māori woman. This led to autonomy over her health and surgical decisions, which assisted with reducing the surgeon-patient power balance:

[My doctors outlook came across as] It’s about the patient actually having control, this is their body. And I’m merely a vessel to help, um to help the patient make the decision that’s best for them in the long run. BC20, age 42, Māori, Mastectomy alone

Whakawhanaungatanga, the process of establishing relationships and relating to each other, was also evident for another participant, who expressed navigating the balance between the wants of her surgical team and her own health choices as part of the shared decision-making process:

So I always came in with information and what I wanted, but I found the surgeon, yes there were bits that he would take on board and he’d go, well this is up to you. But there were other times where he sort of had his own idea of how things are gonna happen and what he wanted from it as well. They were- they laid out all the options. And went you could do this, you could do this, you could do this, or you could do nothing. You could do absolutely nothing. The choice is yours. BC23, age 44, Māori, Mastectomy and implant reconstruction

While shared decision-making is considered ‘best-practice’, in some cases this was not always perceived to be optimally achieved. One woman expressed a significant lack of shared decision-making or agency over her own healthcare, to the point where she was denied surgery (and therefore healthcare) if she decided not to undergo radiation therapy:

Yeah, he [the surgeon] was very, very adamant that it was lumpectomy and yes he definitely did say, if you decide, if you’re going to say no to the radiation, I won’t do the surgery. BC12, age 42, non-Māori, BCS

Some women did not feel comfortable bringing up questions with their surgeon and felt that they were being rushed through the system, potentially hindering the efficacy of ‘shared’ decision-making. As two women described:

It still felt like I’m being intrusive of knowing what’s going on, it, yeah. Which it shouldn’t have felt. I kind of felt nervous even asking questions. BC07, age 45, non-Māori, BCS

And I felt like I was yea, ah, being annoying asking questions. Because he [the surgeon] wanted me out the door. BC12, age 42, non-Māori, BCS

There can be difficulties when attempting to effectively achieve shared decision-making from clinical and patient perspectives of ‘best health outcomes’. One woman described a battle to get a bilateral mastectomy, and described conflict between herself and her surgical team, leading to her feeling unsupported and unheard in her decision:

I had to insist on having the mastectomy. They did not want to do that. And I had to fight because of my family history. BC10, age 50, non-Māori, Mastectomy and implant reconstruction

Another woman described feeling that her healthcare decisions were not in her own hands, suggesting that she did not feel actively involved in the shared decision-making process with her surgeon:

I felt I wasn’t heard. I felt like they had made the decision for me. BC01, age 48, Māori, BCS

### Information and decision-making

Making decisions about surgical options following diagnosis was difficult and experienced differently by all women interviewed. Reactions to cancer diagnosis varied; some women expressed the need for time to process their diagnosis before making decisions and highlighted the need for a range of modes with which to deliver treatment information. The concept of ‘younger’ women with BC was expressed as lacking in much of the information, making the experience unrelatable for some participants.

Many women described a sense of ‘shock’ after their diagnosis which hindered their ability to absorb information obtained in the initial surgical consultation:

I heard the diagnosis, and then I didn’t really hear anything else. And the doctors’ talking to me and I’m sitting there going uh-huh, ok yep, yep, and my husband’s listening and answering questions, and I can’t even tell you what they were because I don’t know. You hear what’s happening, but nothing comes in. BC22, age 45, non-Māori, Mastectomy alone

When you’re first diagnosed, when you’re first run through what’s going through, um, you can’t absorb it all. BC46, age 47, non-Māori, Mastectomy and DIEP reconstruction

One woman expressed that having some time to process her diagnosis was beneficial and helped with her ability to make decisions by gathering information she wanted (through questions) and process what she had been told:

I went back the following week, and I had a list of questions. And I said look, I couldn’t ask on the day because I couldn’t even tell you what my name was. Good, give me time to process the fact you’ve just told me I’ve got stage three breast cancer, thanks. BC22, age 45, non-Māori, Mastectomy alone

For some women, the mode in which information was delivered was important to their experience of decision-making. Some women did not find written information in pamphlets very useful, and felt a sense of ‘overload’ when trying to make decisions, as described by one woman:

It was too much at the beginning, but as I said I didn’t read it all. It can be quite, daunting to start reading something like that [a pamphlet]. BC43, age 48, non-Māori, Mastectomy and DIEP reconstruction

Including different modes such as videos or diagrams was suggested as a useful aid to support decision-making, as two women highlighted:

I’m very much a visual person. So, diagrams and pictures and stuff [are good]. Don’t give me just a paragraph of information. BC12, age 42, non-Māori, BCS

Diagrams are always good you know, but it depends how involved they need to be. But yeah, I guess a video will probably be easier as well, to be fair. You can always go back and listening is a lot more, is a lot more interactive, yea, you can take it in a bit easier. BC43, age 48, non-Māori, Mastectomy and DIEP reconstruction

One wāhine Māori highlighted that much of the BC information is unrelatable to ‘younger’ women, which impacted their decision-making and the need to look to other women as a role model for what they have experienced:

There’s no material out there on young mums. I’m considered medically young to have breast cancer. But there’s a lot of younger women out there that are getting breast cancer. So our publications, our stuff on the website, our posters [in waiting rooms], they’re not reflecting the wider area of breast cancer. BC20, age 42, Māori, Mastectomy alone

Some women went online to find associated groups or relatable communities to assist with their decision-making process, as one woman expressed:

I joined a Facebook group just to read and look at what living flat was going to be kind of like. BC10, age 50, non-Māori, Mastectomy and implant reconstruction

## Discussion

Deciding on BC surgery is a complex, highly individualised decision that is influenced by a number of factors. As for most BC patients, fear and the drive to ‘get the cancer gone’ was a primary influence on decision-making for the younger women interviewed in this study. Shared decision-making was highly valued by most participants, especially for wāhine Māori, and was associated with positive reflections of the cancer experience. An overload of information at the time of diagnosis was a strong theme emerging from interviews. Presenting information via visual formats was suggested as a way to improve information absorption.

Our findings corroborate the existing literature reporting that decision-making is commonly influenced by clinical, physician and personal factors (Gu et al., 2018), as well as other sources, including friends, family and BC survivors (Chang et al., 2016; Rosenberg et al., 2018, 2019). For the current cohort, the primary, initial factor driving decision-making was that of fear and survival, where ‘life over breasts’ and ‘getting it gone’ were commonly expressed themes emerging from interviews. Fear is a common emotion associated with a cancer diagnosis (Mazzocco et al., 2019) where peace of mind is often a key factor in younger women’s decision-making (Rosenberg et al., 2018). For some women interviewed, mastectomy was the most extreme measure to offer peace of mind and mitigate fear of cancer recurrence. Women often choose mastectomy as the most reassuring option (Gu et al., 2018), and where mastectomy was clinically indicated, or accompanied by a known family history, deciding on mastectomy with their surgeon’s support represented low decisional conflict for interviewees. For younger women where BCS was the preferred option, the need to ‘get it gone’ for peace of mind was just as prevalent; these women also wanted surgery as fast as possible to alleviate worries of the cancer ‘growing’ and spreading further. Again, where BCS was clinically indicated, women reported no apparent distress in making this choice.

However decisional conflict could arise if a surgeon’s recommendation did not align with a woman’s preference for surgery. Moreover, that preference could be influenced by the advice or experiences of friends or family who had been through BC surgery. Friends, family and BC survivors are often important sources of information for newly diagnosed women (Rosenberg et al., 2018). In particular, women in the current cohort with mothers who may have experienced psychological distress following either mastectomy or BCS were often influenced by their experience and level of surgical satisfaction. Traumatic or other experience with BC can have implications for BC prevention decisions (Padamsee et al., 2020). Our findings are suggestive that the experience of friends and family has implications for treatment decisions also. Body image and how women viewed their breasts was part of decision-making but did not appear to be a major influencing factor for this cohort. This is similar to Rosenberg et al. (2019) who reported cosmetic appearance (cosmesis) as a concern for some younger women, but in contrast to Paterson et al. (2016) who reported that body image was a frequent concern for younger women diagnosed with BC. Women in the current study reported wanting to have the primary surgery first and deal with that [reconstruction] later, again reiterating that thoughts of survival and getting the cancer gone were a primary concern. Timing of interviews may be the source of any differences. It is possible that the younger women interviewed did not report body image as a key influence on surgical decisions because they were interviewed post-surgery and so were providing a retrospective viewpoint of their experience. Perhaps if we had interviewed immediately post-diagnosis, but prior to surgery, concerns about cosmesis may have been more evident.

While cosmesis was not a key factor for this group, younger women still appraised their breasts and feminine identity. For example, women often stated that they were ‘still young enough’ and ‘too young to not have something there’ when considering breast reconstruction. This aligns with Soon et al. (2019) who noted younger women as being more likely to opt for reconstructive surgery. Interestingly for this group, three of the seven women (42.9%) who underwent mastectomy did not have reconstruction and chose to remain flat. This reflects an increasing interest in ‘going flat’ (i.e. bilateral mastectomy without reconstruction) (Kritz, 2022) and represents an interesting shift against societal and culturally created norms about breasts and their importance for, and to, women and feminine identity (La et al., 2019; Webb et al., 2019). With more women choosing to go flat, medical teams need to discuss this an option (Baker et al., 2021), and should not assume that it is an undesirable option for all women (Holland et al., 2016). Recent research reports that information on this option is not routinely provided to women during decision-making (Wakeley et al., 2020). For example, a recent US survey of women who decided against breast reconstruction following mastectomy reported that for 22% of these women, surgeons did not offer the option to go flat (Baker and Attai, 2021). Another US survey reported that 38% of women felt unsupported by their surgical team and had to research going flat for themselves (Wakeley et al., 2020). Our findings somewhat support this evidence, with one interviewee expressing feeling unsupported in her decision, despite severe post-reconstruction complications behind her reasoning for going flat. Going flat has implications for aesthetic surgical closure (Morrison and Karp, 2022) and may be a delicate discussion for surgeons, who need to balance emotional reactions to a cancer diagnosis with fully informing women of all surgical options before they embark on mastectomy as a radical treatment that is irreversible.

A second theme evident in the interviews concerned the sense of autonomy the shared decision-making process facilitated for women considering treatment. The quality of the shared decision-making process was pivotal prior to surgery and was especially important for wāhine Māori, who valued the establishment of whakawhanaungatanga (relationality). Patients also typically appraise their interactions with health care professionals as either positive or negative (Marcinowicz and Górski, 2016), a rating that can be influenced by the quality of clinician communication (Blackmore et al., 2021) and interpersonal skills (Blackmore et al., 2020). Many of the women interviewed were engaged in shared decision-making and subsequently reported feeling in control of what was happening to their bodies, which led to a more positive experience overall and a positive appraisal of their surgical outcome. These findings align with those of Clarke et al. (2021) who also noted that patients engaged in shared decision-making reported positive outcomes. To further illustrate this point, the few women who did not report good shared decision-making cited feeling that they had not been listened to, that they felt forced into surgical recommendations and were being ‘annoying’ when asking too many questions. These women tended to reflect negatively on the shared decision-making process and for some, reported lower satisfaction with their surgical result and a lack of autonomy over their own body.

The final theme evident in the interviews involved the role of information in decision-making. Women reported receiving good information, although recognised that it was both overwhelming and difficult to absorb it at the time of delivery (typically at point of diagnosis) due to high levels of distress on hearing the word ‘cancer’. Shock and distress on receiving a cancer diagnosis is commonly experienced by cancer patients and is most evident in the period following diagnosis (Fortin et al., 2021). Diagnostic distress associated with anxiety and fear can impact thinking (Mazzocco et al., 2019) and dominate decision-making (Brown et al., 2017). Post diagnostic distress was certainly reported by all interviewees in the current cohort. This may have implications for the shared decision-making process (Keij et al., 2021), where clinicians must recognise that patients may initially be too shocked to effectively participate in decision-making (Brown and Salmon, 2019).

For patients who do want to engage in shared decision-making, there is gathering evidence that the multi-modal presentation of medical information improves patient understanding. For example, Meppelink et al. (2015) showed the effectiveness of presenting complex medical information via visual animation combined with spoken text, especially for individuals with low health literacy. Similarly, Glassey et al. (2018) suggested that photos may help younger women undergoing breast reconstruction manage their expectations about how reconstructed breasts might look following surgery. Several researchers have shown the effectiveness of pictorial decision aids in facilitating patient comprehension and decision-making (e.g. Alam et al., 2016; Durand et al., 2016, 2021). Younger women want visual information depicting their surgical options, and specifically want tailored, age-specific information (Recio-Saucedo et al., 2018). Showing good visual examples may be a particularly important resource for those women who do want to explore the option of going flat (Wakeley et al., 2020). Supporting this evidence, women in the current cohort highly valued their surgeon drawing pictures or diagrams to depict how their breasts might look following surgery, which did help to alleviate pre-surgery worry. For wāhine Māori, a surgeon sharing even the most basic of hand drawn diagrams helped to establish whakawhanaungatanga. Women also noted that the posters and information booklets at clinics did not depict younger women with BC, so felt that this was ‘not reflecting the wider area of BC’.

The findings reported here build on the existing knowledge base outlining factors involved in decision-making in younger women diagnosed with BC. They also signify the only recent patient-focused investigation of surgical decision-making in younger NZ women. While qualitative research does not seek generalisability, a key strength of qualitative research is that it allows participants to speak about their experience from their perspective, which allowed Māori participants, who represent a unique population within NZ, to contribute their stories in a culturally safe space with analysis by Māori researchers. However, while three researchers identified as Māori, this study design was not derived from an Indigenous worldview. Future research conducted from a Kaupapa Māori approach (Smith, 2021) could extend the findings of this study for Māori decision-making, which may, for example, explore the role of Rongoa Māori (traditional healing systems) in the decision-making process. All interviewers were female but had no direct lived experience of BC surgery apart from family and friends who had experienced BC. Including a research team member in future research with lived experience could be beneficial. However, the research team had extensive qualitative experience with cancer participants, which was a strength for this study, reducing any potential power imbalance and enabling deeper relatability with participants during interviews. A further limitation involved COVID-19 constraints, where interviews were restricted to telephone or Zoom video call only. This impacted the establishment of a level of rapport usually afforded by a more personal one-on-one experience. Women interviewed provided comments that were very much reflective of their surgical experience. If data had been collected following diagnosis and pre-surgery, we may have captured a different perspective and identified varying levels of decisional conflict as women processed their diagnosis and treatment decisions. Future directions should investigate how women from other regions in NZ experience BC and determine if there is any regional variation between rural and urban centres. There should also be an investigation into going flat following mastectomy, and the incidence of treatment regret in those who might choose this option.

### Conclusion

Surgical decisions for younger women diagnosed with BC are guided by multiple factors, the primary of these being survival: needing to act to ‘get the cancer gone’. Shared decision-making was highly valued by both Māori and non-Māori women as an important contributor to support women in their decisions, where a good patient-clinician relationship facilitated a sense of autonomy over women’s bodies and health decisions, and ultimately led to a more positive appraisal of the BC experience. Good information is key to shared decision-making, and women particularly expressed a need for multi-modal presentation of information and material reflecting younger women with BC.
